# The influence of physical performance on technical and tactical outcomes in the UEFA Champions League

**DOI:** 10.1186/s13102-022-00573-4

**Published:** 2022-10-10

**Authors:** Toni Modric, James J. Malone, Sime Versic, Marcin Andrzejewski, Paweł Chmura, Marek Konefał, Patrik Drid, Damir Sekulic

**Affiliations:** 1 Faculty of Kinesiology, University of Split, Split, Croatia; 2 School of Health and Sport Sciences, Liverpool Hope University, Liverpool, UK; 3 Department of Methodology of Recreation, Poznań University of Physical Education, Poznań, Poland; 4 Department of Team Games, Wrocław University of Health and Sport Sciences, Wrocław, Poland; 5 Department of Human Motor Skills, Wrocław University of Health and Sport Sciences, Wrocław, Poland; 6 Faculty of Sport and Physical Education, University of Novi Sad, Novi Sad, Serbia

**Keywords:** Football, Running performance, Technical-tactical performance, Success, Linear mixed model

## Abstract

**Background:**

To clarify does physical performance affect success in highest-level soccer, the purpose of the present study was to identify differences in technical-tactical performance (TP) between teams covering high and low running performance (RP) during the UEFA Champions League (UCL) matches.

**Methods:**

The RP and TP data were collected from UCL group stage matches in the 2020/21 season. RP variables included total distance covered (TD), high intensity running (HIR), total distance when in ball possession (TDB), and high intensity running when in ball possession (HIRB). TP variables included goal chances, shots, shots on target, passes, accurate passes, key passes, key passes accurate, crosses, crosses accurate, counter attacks, counter attacks with a shot, high pressing, high pressing successful, low pressing, low pressing successful, tackles, tackles successful, entrances to the opponent’s box, total actions, and successful actions. K-means cluster analysis method was used to classify teams covering (i) low and high TD, (ii) low and high HIR, (iii) low and high TDB, (iv) low and high and HIRB. Linear mixed models were used to identify differences in teams’ TP according to their RP. Pearson’s correlations were used to establish direct association between team TP and RP.

**Results:**

Similar TP were observed whether teams covering high or low TD/HIR. Teams covering greater TDB/HIRB had more goal chances, shots, shots on target, passes, accurate passes, key passes, accurate key passes, crosses, successful high pressing, entrances to the opponent’s box, total actions, and successful actions were observed (all moderate to very large effect sizes. Significant association between specific TP variables and TDB/HIRB were evidenced (Pearson’s r = 0.35–0.96, all *p* < 0.05).

**Conclusion:**

Covering greater TDB and HIRB may allow more frequent execution of fundamental TP which are considered essential for match success, indicating that RP when team has ball in possession is important determinant of success in highest-level soccer. This study shows that physical performance affect success in highest-level soccer.

## Background

Soccer is a multifactorial, complex sport that requires high levels of technical, tactical, physical and psychosocial qualities [1, 2]. Successful match performance is highly dependent on the interaction of these factors [3, 4]. Interestingly, although technical-tactical performance (TP) are considered decisive for success in soccer [5, 6], physical performance (i.e., quantified by running performance [RP] such as total distance covered and distances covered in various speed zones) are more commonly investigated [3, 7, 8]. Such growing interest in RP has led to a large body of published research [3, 9, 10]. Although this has forcibly shaped contemporary opinions, with researchers and practitioners frequently emphasizing the importance of RP, particularly high-intensity running, in professional soccer [9, 11], current research remains equivocal regarding the high-intensity distance covered and success in soccer. For example, some research has demonstrated that soccer players perform significantly lower amounts of high-intensity running when winning than when compared to losing situations [12–15]. Conversely, other studies report no differences in high-intensity running regardless of the match outcome [16–18]. In addition, older research reported that players from lower-ranked teams performed a greater amount of high-intensity running than their counterparts on better teams [5, 19], while recent research has revealed no differences in high-intensity running irrespective to final competition position [20, 21].

Evident inconsistencies in these findings indicate that utilizing success indicators such as match outcome and position on the table may be controversial to clarify does RP affect success in soccer. On the other hand, TP, such as passes and shots, are considered as essential for match success in professional soccer [22–27]. Such measures may differ between teams depending on the level of success. Previous research has suggested that top-ranked teams have greater amounts of ball possession in the opponent’s half, number of passes in the final third of the field and overall time in possession [28, 29]. Furthermore, significant differences in total shots, shots on target and crosses were evidenced between successful and unsuccessful teams [30].

As these reports clearly indicate TP as important determinants of success in soccer [22, 30, 31], TP could be valid success indicator. Thus, analysing TP of teams covering high and low match RP may clarify does physical performance affect success in soccer. However, given that currently such studies lacking, more research is needed. The results of such research may provide new knowledge, enabling a detailed understanding of the relationship between TP and RP, while at the same time refining prior knowledge about success in soccer. Therefore, the purpose of the present study was to identify differences in TP between teams covering high and low RP during the UEFA Champions League (UCL) matches. Initially, we hypothesized that teams covering high RP at high-intensity will be more successful (i.e., will achieve greater TP).

## Methods

This investigation was conducted in several phases. Firstly, we tested TPs to confirm them as valid benchmarks of success in the knockout stage of the UCL. Secondly, we classified teams according to their RP (low/high). Thirdly, we analysed differences in TP between teams covering high and low RP, and confirmed results furtherly by analysing direct association between TP and RP variables.

### Sample

The sample comprised 547 individual match observations of 378 outfield players (goalkeepers were excluded due to the specificities of position) which were members of 24 teams that competed in the group stage of the UCL in the 2020/21 season. All data were obtained from 20 matches from groups A (n = 3), B (n = 3), C (n = 4), E (n = 4), F (n = 3), and G (n = 3). To identify association between teams’ RP and TP, individual players’ performances were jointly evaluated into the teams’ performances, and used as cases in this study.

All data were anonymized in accordance with the principles of the Declaration of Helsinki to ensure player and team confidentiality. The investigation was approved by the local university ethics board (approval number: 2181-205-02-05-19-0020). As investigation is anonymous and include adult participants, informed consent was waived. Written permission for data used was obtained from Instat Limited (Limerick, Republic of Ireland, 5 June 2021).

### Procedures

All data were recorded using a multicamera, semi-automatic optical tracking system (InStat Fitness, Instat Limited, Limerick, Republic of Ireland). This system includes 3 static cameras (i.e., 2 × Full HD and 1 × 4 K camera) installed on the roof of the soccer stadium. The system has a sampling frequency of 25 Hz, and identifies players by their movement, shape, and color information. It has previously received ‘FIFA Quality’ status as part of the test protocol for Electronic and Performance Tracking Systems (EPTS) validation (authorization number: 1007382), demonstrating high levels of absolute and relative reliability [32]. A detailed report is available on the official FIFA webpage.

We observed the overall RP and RP when the team was in ball possession. The variables included total distance (TD), high-intensity running (> 19.8 km/h) (HIR), total distance when in ball possession (TDB), and high-intensity running when in ball possession (> 19.8 km/h) (HIRB). The TP variables and associated definitions are presented in Table 1.

**Table 1:** Technical-tactical performance variables and their definitions

Goal chances	Number of created goal chances, finished with a shot or without a shot
Shots	Total number of shots to score
Shots on target	Number of shots within a goal
Passes	Total number of passes
Accurate passes	Total number of accurate passes
Key passes	Passes to a partner who is in a goal scoring position (i.e., one-on-one situation, empty net, etc.) And passes to a partner that “cuts off” the whole defensive line of the opponent’s team (3 or more players) in the attacking phase
Key passes accurate	Total number of accurate key passes
Crosses	Number of long passes performed by a player from an offensive zone (last 40 m of pitch between the short side of the penalty area and the lateral side of the field) and directly to the penalty area
Crosses accurate	Total number of accurate crosses
Counter attacks	Open play attack after the opponent team loses the ball (a counterattack lasts no longer than 20 s, and the speed of moving to the target in a counterattack is not less than 3 m/s)
Counter attacks with a shot	Counterattacks that include shots toward the goal
High pressing	Total number of collective attempts to force the opponents to lose the ball or to stop the development of an attack on the opponent’s half of the pitch
High pressing success	Number of successfully performed high pressings
Low pressing	Total number of collective attempts to force the opponents to lose the ball or to stop the development of an attack on one’s own half of the pitch
Low pressing success	Number of successfully performed low pressings
Tackles	Active action of a player who tries to tackle the ball from the player possessing it
Tackles successful	Total number of successful tackles
Entrances to the opponent’s box	Number of entries into the opponent’s penalty area
Total actions	Total number of all types of actions (including passes, crosses, set pieces passes, tackles, challenges, shots, etc.)
Successful actions	Total number of successful actions

### Statistical analyses

The Kolmogorov–Smirnov test revealed that all data were normally distributed (all K-S *p* > 0.05). Homogeneity was confirmed using Levene’s test. The statistical analyses were performed throughout several phases. As a preliminary analysis, we correlated TP and total group points at the end of the group stage to evaluate the validity of TP variables as benchmark of success in the UCL. For this purpose, we performed multiple regression analysis with TP as a predictor and total group points at the end of the UCL group stage as the criterion. We computed and reported multiple correlations (multiple R) and coefficients of determination (R^2^).

Next, we classified teams into the two groups (low performance/high performance) according to their RP using the k-means cluster analysis method [33]. Two clusters for each RP variable identified teams covering (i) low and high TD, (ii) low and high HIR, (iii) low and high TDB, (iv) low and high and HIRB.

Afterwards, a series of linear mixed models were developed to identify differences in team TP according to their RP, considering the team identity to account for repeated measures (i.e., random effect). The 95% confidence intervals were computed to assess the precision of the estimates. The t-statistics from the mixed models were converted into Cohen’s d effect sizes [34] and interpreted as < 0.2, trivial; 0.2–0.6, small; 0.6–1.2, moderate; 1.2–2.0, large; > 2.0, very large [35].

Finally, Pearson’s correlation coefficients were computed to establish direct association between team TP and RP, With the r coefficient classification as previously suggested: r < 0.35 indicates a low or weak correlation, r = 0.35 to 0.67 denotes a modest or moderate correlation, r = 0.68 to 0.9 implies a strong or high correlation, and r > 0.90 refers to a very high correlation [36]. All analyses were performed using SPSS software (IBM, SPSS, Version 25.0), and the significance level was set to *p* < 0.05.

## Results

The TP variables as predictors were significantly correlated with total points earned at the end of the group stage as criteria, demonstrating the appropriate validity of herein studied TP as a benchmark of success in the group stage of the UCL. In brief, the predictors explained 71% of the criterion’s variance (Table 2).

**Table 2:** Multiple regression calculation for total points earned at the end of the group stage of UCL

	β	Std.Err. β	B	Std.Err. B	t	*p*
*Intercept*			10.85	8.19	1.33	0.20
Goal chances	− 0.72	0.48	− 1.06	0.70	− 1.51	0.15
Shots	0.41	0.38	0.42	0.39	1.08	0.29
Shots on target	0.08	0.28	0.16	0.59	0.28	0.78
Passes	− 0.31	4.93	− 0.01	0.17	− 0.06	0.95
Accurate passes	2.30	5.44	0.08	0.20	0.42	0.68
Key passes	0.79	0.39	0.93	0.46	2.02	0.06
Key passes accurate	− 0.16	0.50	− 0.33	1.00	− 0.33	0.74
Crosses	− 0.17	0.36	− 0.11	0.22	− 0.47	0.64
Crosses accurate	− 0.33	0.30	− 0.56	0.51	− 1.10	0.29
Counterattacks	0.12	0.23	0.14	0.26	0.52	0.61
Counter attacks with a shot	0.20	0.19	0.09	0.08	1.07	0.30
High pressing	− 0.34	0.36	− 0.39	0.41	− 0.95	0.35
High pressing successful	0.43	0.40	0.71	0.64	1.10	0.29
Low pressing	− 0.38	0.25	− 0.43	0.28	− 1.55	0.14
Low pressing successful	− 0.14	0.26	− 0.30	0.56	− 0.53	0.60
Tackles	− 0.33	0.19	− 0.23	0.13	− 1.70	0.11
Tackles successful	**0.59**	**0.24**	**0.76**	**0.31**	**2.49**	**0.02**
Entrances to the opponent’s box	0.27	0.40	0.19	0.28	0.69	0.50
Total actions	− 0.26	2.60	− 0.01	0.09	− 0.10	0.92
Successful actions	− 1.54	3.54	− 0.05	0.12	− 0.44	0.67
R	0.84					
R^2^	0.71					
*p*	0.03					

*Intercept* interception coefficient, *β* standardized regression coefficient, *B* non-standardized regression coefficient, *R* coefficient of the multiple correlation, *R2* coefficient of determination

Bold text denotes statistical significance of *p* < 0.05

Average values of teams covering low (L-TD) and high (H-TD) TD were 113,105 ± 2797 m (range 105,653–116,482 m, n = 22) and 120,141 ± 2332 m (range 116,991–125,258 m, n = 18), respectively. Average values of teams covering low (L-HIR) and high (H-HIR) HIR were 8565 ± 631 m (range 6995–9327 m, n = 17) and 10,321 ± 761 m (range 9541–12,200 m, n = 23), respectively.

Average values of teams covering low (L-TD) and high (H-TD) TDB were 30,613 ± 4613 m (range 23,281–37,593 m, n = 19) and 46,359 ± 5806 m (range 39,132–63,098, n = 21), respectively. Average values of teams covering low (L-HIR) and high (H-HIR) HIRB were 3034 ± 335 m (range 2203–3650 m, n = 23) and 4388 ± 675 m (range 3739–5736 m, n = 17), respectively.

There were no significant differences in any TP variable between the H-TD and L-TD teams. Additionally, there were no significant differences in TP between the H-HIR and L-HIR teams (Table 3).

**Table 3:** Differences in technical-tactical performance according to the running performance

	Total distance			High intensity running		
	B (SE)	Lower CI–Upper CI	Effect size	B (SE)	Lower CI–Upper CI	Effect size
Goal chances	0.31 (1.08)	− 1.87 to 2.5	0.10 (trivial)	0.97 (1.04)	− 1.15 to 3.08	0.33 (small)
Shots	0.75 (1.52)	− 2.34 to 3.83	0.17 (trivial)	2.09 (1.47)	− 0.89 to 5.06	0.48 (small)
Shots on target	− 0.27 (0.72)	− 1.72 to 1.19	− 0.13 (trivial)	1.16 (0.68)	− 0.22 to 2.54	0.61 (moderate)
Passes	− 2.16 (33.55)	− 71.09 to 66.77	− 0.02 (trivial)	1.62 (31.64)	− 63.65 to 66.9	0.02 (trivial)
Accurate passes	3.7 (31.17)	− 60.53 to 67.93	0.05 (trivial)	5.9 (29.35)	− 54.82 to 66.63	0.08 (trivial)
Key passes	0.00 (1.44)	− 2.92 to 2.91	0.00 (trivial)	0.76 (1.4)	− 2.09 to 3.6	0.18 (trivial)
Key passes accurate	− 0.2 (0.86)	− 1.94 to 1.54	− 0.08 (trivial)	0.70 (0.84)	− 0.99 to 2.4	0.28 (small)
Crosses	− 0.67 (2.15)	− 5.08 to 3.74	− 0.12 (trivial)	3.73 (1.97)	− 0.32 to 7.78	0.76 (moderate)
Crosses accurate	− 0.07 (0.68)	− 1.47 to 1.33	− 0.05 (trivial)	0.4 (0.64)	− 0.94 to 1.75	0.28 (small)
Counter attacks	− 0.28 (1.49)	− 3.29 to 2.73	− 0.06 (trivial)	1.91 (1.43)	− 0.99 to 4.81	0.44 (small)
Counter attacks with a shot	2.74 (3.89)	− 5.21 to 10.68	0.26 (small)	− 3.21 (3.61)	− 10.66 to 4.23	− 0.36 (small)
High pressing	− 0.4 (1.47)	− 3.37 to 2.58	− 0.09 (trivial)	1.47 (1.41)	− 1.39 to 4.33	0.36 (small)
High pressing successful	− 0.81 (1.04)	− 2.91 to 1.3	− 0.25 (small)	0.57 (1.02)	− 1.49 to 2.64	0.19 (trivial)
Low pressing	2.24 (1.45)	− 0.72 to 5.21	0.59 (small)	1.04 (1.49)	− 1.99 to 4.07	0.23 (small)
Low pressing successful	− 0.17 (0.80)	− 1.80 to 1.45	− 0.07 (trivial)	− 0.14 (0.79)	− 1.75 to 1.47	− 0.06 (trivial)
Tackles	0.16 (2.45)	− 4.8 to 5.12	0.02 (trivial)	− 3.16 (2.41)	− 8.04 to 1.72	− 0.43 (small)
Tackles successful	0.77 (1.3)	− 1.89 to 3.43	0.22 (small)	0.43 (1.3)	− 2.21 to 3.07	0.11 (trivial)
Entrances to the opponent’s box	0.92 (1.95)	− 3.07 to 4.92	0.18 (trivial)	1.78 (1.89)	− 2.09 to 5.65	0.35 (small)
Total actions	22.44 (37.68)	− 54.76 to 99.64	0.23 (small)	− 3.89 (36.02)	− 77.95 to 70.18	− 0.04 (trivial)
Successful actions	22.98 (34.06)	− 47.16 to 93.12	0.27 (small)	− 2.01 (32.52)	− 69.22 to 65.2	− 0.03 (trivial)

*B *estimate, *SE* standard error, *CI *confidence interval

We noted significant differences in TP variables between the H-TDB and L-TDB teams, and between the H-HIRB and L-HIRB teams. The H-TDB and H-HIRB teams had greater number of goal chances (4.2 and 4.34, respectively), shots (6.93 and 5.03, respectively), shots on target (2.49 and 2.72, respectively), passes (160 and 127, respectively), accurate passes (142 and 114, respectively), key passes (4.03 and 3.92, respectively), accurate key passes (1.89 and 2.65, respectively), crosses (7.76 and 4.99, respectively), successful high pressings (2.96 and 2.56, respectively), entrances to the opponent’s box (8.95 and 9.34, respectively), total actions (168 and 158, respectively), and successful actions (150 and 140, respectively) (all moderate to very large effect sizes) (Table 4).

**Table 4:** Differences in technical-tactical performance according to the running performance with ball in possession

	Total distance with ball in possession			High intensity running with ball in possession		
	B (SE)	Lower CI–Upper CI	Effect size	B (SE)	Lower CI–Upper CI	Effect size
Goal chances	**4.2 (0.92)**	**2.26 to 6.14**	2.18 (very large)	**4.34 (1.00)**	**2.31 to 6.36**	1.41 (large)
Shots	**6.93 (1.2)**	**4.49 to 9.36**	1.91 (large)	**5.03 (1.47)**	**2.04 to 8.02**	1.16 (moderate)
Shots on target	**2.49 (0.7)**	**1.08 to 3.91**	1.21 (large)	**2.73 (0.69)**	**1.33 to 4.12**	1.29 (large)
Passes	**160.09 (29.86)**	**99.09 to 221.1**	1.97 (large)	**126.87 (37.11)**	**51.73 to 202.01**	1.11 (moderate)
Accurate passes	**142.39 (29.66)**	**81.64 to 203.14**	1.81 (large)	**113.68 (35.71)**	**41.29 to 186.06**	1.05 (moderate)
Key passes	**4.03 (1.25)**	**1.5 to 6.57**	1.04 (moderate)	**3.92 (1.38)**	**1.07 to 6.77**	1.17 (moderate)
Key passes accurate	**1.89 (0.77)**	**0.34 to 3.44**	0.80 (moderate)	**2.65 (0.81)**	**0.95 to 4.34**	1.53 (large)
Crosses	**7.76 (2.06)**	**3.56 to 11.97**	1.39 (large)	**4.99 (2.47)**	**0.02 to 10**	0.66 (moderate)
Crosses accurate	1.09 (0.78)	− 0.51 to 2.7	0.58 (small)	**2.00 (0.8)**	**0.36 to 3.64**	0.87 (moderate)
Counter attacks	0.13 (1.53)	− 3 to 3.26	0.03 (trivial)	2.46 (1.52)	− 0.64 to 5.55	0.59 (small)
Counter attacks with a shot	0.85 (4.25)	− 7.79 to 9.5	0.07 (trivial)	2.34 (4.44)	− 6.65 to 11.34	0.17 (trivial)
High pressing	2.28 (1.53)	− 0.81 to 5.38	0.50 (small)	2.02 (1.57)	− 1.17 to 5.2	0.44 (small)
High pressing successful	**2.96 (0.93)**	**0.98 to 4.93**	1.60 (large)	**2.56 (0.97)**	**0.5 to 4.62**	1.30 (large)
Low pressing	2.34 (1.46)	− 0.68 to 5.38	0.69 (moderate)	− 1.97 (1.56)	− 5.16 to 1.23	− 0.48 (small)
Low pressing successful	**1.84 (0.71)**	**0.39 to 3.30**	0.83 (moderate)	− 0.52 (0.82)	− 1.74 to 1.63	− 0.02 (trivial)
Tackles	− 1.43 (2.43)	− 6.35 to 3.48	− 0.19 (trivial)	− 3.49 (2.4)	− 8.34 to 1.37	− 0.47 (small)
Tackles successful	1.91 (1.26)	− 0.63 to 4.45	0.49 (small)	− 0.03 (1.31)	− 2.75 to 2.7	− 0.01 (trivial)
Entrances to the opponent’s box	**8.95 (1.82)**	**5.27 to 12.63**	1.62 (large)	**9.34 (1.73)**	**5.83 to 12.85**	1.80 (large)
Total actions	**168.18 (35.09)**	**96.86 to 239.5**	1.65 (large)	**158.28 (39.34)**	**78.62 to 237.93**	1.31 (large)
Successful actions	**150.12 (33.45)**	**81.78 to 218.47**	1.65 (large)	**140 (37.86)**	**63.33 to 216.67**	1.31 (large)

*B* estimate, *SE* standard error, *CI* confidence interval

Bold text denotes statistical significance of *p* < 0.05

Table 5 presents direct associations between the teams’ RP and TP. We did not observe any correlations between TD and TP (all *p* > 0.05). HIR in general was not strongly related to the TP. More precisely, of 21 correlations, only two reached statistical significance, with shots and accurate crosses being positively correlated with HIR (both approximately 15% of common variance).

**Table 5:** Associations between technical-tactical performance and running performance (data are given as r (*p*))

	Running performance		Running performance with ball in possession	
	TD	HIR	TDB	HIRB
Goal chances	0.06 (0.694) (low)	0.27 (0.086) (low)	**0.59 (0.001) (moderate)**	**0.51 (0.001) (moderate)**
Shots	0.23 (0.162) (low)	**0.39 (0.012) (moderate)**	**0.68 (0.001) (high)**	**0.50 (0.001) (moderate)**
Shots on target	0.04 (0.822) (low)	0.23 (0.161) (low)	**0.63 (0.001) (moderate)**	**0.46 (0.003) (moderate)**
Passes	− 0.01 (0.97) (low)	0.12 (0.466) (low)	**0.96 (0.001) (very high)**	**0.49 (0.001) (moderate)**
Accurate passes	0.02 (0.888) (low)	0.11 (0.483) (low)	**0.96 (0.001) (very high)**	**0.48 (0.002) (moderate)**
Key passes	− 0.01 (0.954) (low)	0.19 (0.253) (low)	**0.49 (0.001) (moderate)**	**0.35 (0.033) (moderate)**
Key passes accurate	0.04 (0.809) (low)	0.20 (0.228) (low)	**0.42 (0.007) (moderate)**	**0.35 (0.036) (moderate)**
Crosses	− 0.06 (0.718) (low)	0.20 (0.206) (low)	**0.64 (0.001) (moderate)**	0.27 (0.094) (low)
Crosses accurate	0.07 (0.65) (low)	**0.38 (0.015) (moderate)**	**0.58 (0.001) (moderate)**	**0.42 (0.007) (moderate)**
Counter attacks	0.10 (0.534) (low)	0.22 (0.169) (low)	− 0.04 (0.83) (low)	0.29 (0.065) (low)
Counter attacks with a shot	− 0.03 (0.837) (low)	− 0.10 (0.531) (low)	0.10 (0.556) (low)	− 0.02 (0.886) (low)
High pressing	− 0.01 (0.939) (low)	0.28 (0.078) (low)	**0.35 (0.028) (moderate)**	0.11 (0.486) (low)
High pressing successful	− 0.10 (0.539) (low)	0.26 (0.103) (low)	**0.42 (0.006) (moderate)**	0.20 (0.225) (low)
Low pressing	0.02 (0.88) (low)	0.17 (0.27) (low)	0.09 (0.56) (low)	− 0.06 (0.69) (low)
Low pressing successful	− 0.26 (0.10) (low)	0.10 (0.52) (low)	**0.35 (0.02) (moderate)**	0.05 (0.73) (low)
Tackles	− 0.01 (0.952) (low)	− 0.31 (0.05) (low)	− 0.15 (0.371) (low)	− 0.18 (0.256) (low)
Tackles successful	0.01 (0.934) (low)	0.07 (0.652) (low)	0.14 (0.405) (low)	0.07 (0.65) (low)
Entrances to the opponent’s box	0.03 (0.863) (low)	0.29 (0.071) (low)	**0.80 (0.001) (high)**	**0.54 (0.001) (moderate)**
Total actions	0.07 (0.67) (low)	0.10 (0.53) (low)	**0.93 (0.001) (very high)**	**0.50 (0.001) (moderate)**
Successful actions	0.09 (0.60) (low)	0.10 (0.54) (low)	**0.94 (0.001) (very high)**	**0.50 (0.001) (moderate)**

Bold text denotes statistical significance of *p* < 0.05

On the other hand, TDB and HIRB were positively correlated with goal chances (both moderate correlations), shots (strong and moderate correlations, respectively), shots on target (both moderate correlations), passes (very strong and moderate correlations, respectively), accurate passes (very strong and moderate correlations, respectively), key passes (moderate and small correlations, respectively), accurate key passes (moderate and small correlations, respectively), entrances to the opponent’s box (strong and moderate correlations, respectively), total actions (very strong and moderate correlations, respectively), and successful actions (strong and moderate correlations, respectively). In addition, high pressings, successful high pressings, and low pressings were correlated with TDB (small to moderate correlations).

## Discussion

This study aimed to identify differences in TP between teams covering high and low RP during the UCL matches. Results indicated that the teams’ TP was not affected by their overall RP (i.e., TD and HIR). On the other hand, we demonstrated significant influence of TDB and HIRB on specific TP, highlighting the importance of the physical performance of the entire team when have ball in possession.

The results of previous studies which investigated physical performance and success indicators are not consistent, implying limited and confusing knowledge about the influence of RP on success in soccer [5, 12–14, 16, 18–21]. However, our results provide new evidence that may explain this issue in detail. Namely, we observed no significant differences in TP between the L-TD and H-TD teams. Additionally, we did not find differences in TP between the L-HIR and T-HIR teams. Further, we did not detect significant correlations between TP and TD. Also, HIR in general was not strongly related to TP. More precisely, of 21 correlations, only two reached statistical significance, with shots and accurate crosses being positively correlated with HIR (both approximately 15% of common variance).

This most specifically means that, irrespective of the TD covered and the HIR of the teams, soccer teams that competed in the UCL had similar numbers of goal chances, shots, passes, key passes, crosses, counterattacks, pressings, tackles, and entrances to the opponent’s box. Moreover, no differences in these successfully performed TPs were found regardless to the teams’ accumulated TD and HIR. Finally, UCL teams achieved a similar number of total actions and total successful actions, irrespective of the TD coverage and HIR of their teams. Such findings clearly indicate that overall RP does not affect TP at the highest level of soccer competition. Since TP are considered essential for match success in professional soccer [22, 24–27, 37], while considering that even findings from our study demonstrates TP variables as benchmarks of success (i.e., herein studied TP variables explained 71% of the variance in total points earned at the end of the group stage), these findings ultimately suggest that the success of highest-level soccer teams is not affected by their overall RP.

Taken together, these findings support prior considerations that overall technical and tactical effectiveness probably have a greater impact on success in soccer than pure physical performance [9]. However, when we analyzed TP variables in relation to RP when teams had ball possession, our results revealed contrasting findings. Specifically, we discovered that the H-TDB and H-HIRB teams had a greater number of shots, passes, key passes, and crosses. In addition, these TP variables were significantly correlated with TDB and HIRB (all moderate to very strong correlations), underlining the important effect of teams’ physical performance in attacking phase of game (i.e., when team had ball possession) on their technical performance. Thus, it seems that covering large TD and HIR in attacking phase of game enabled more frequent execution of fundamental soccer skills such as passing, shooting or crossing [38]. Most likely, such players’ greater activeness (i.e., covering large TD and HIR) in the attacking phase of the game leading to defensive imbalances of opponent team. This playing principal opens more free spaces at the pitch [39], what may enable their teammates more solutions to pass, cross or shoot. Additionally, as we evidenced that H-TDB and H-HIRB teams had a greater number of goal chances and entrances to the opponent’s box, such team behavior (i.e., greater team activeness in the attacking phase of the game) most likely contributed in achieving greater tactical performance as well.

More importantly, it seems that such team behavior may even have a critical influence, not only on more frequent execution of fundamental soccer skills, but on successfully performed key skills that influence match outcome. Namely, we evidenced that H-TDB and H-HIRB achieved a greater number of shots or targets, successful passes, accurate key passes and successful high pressings than the L-TDB and L-HIRB teams (all moderate to large effect sizes). Such results clearly show that, when in ball possession, team RP have important influence on successful execution of specific TP, which are in general considered as crucial for achieving success in matches [22, 24–27, 37]. In view of this, TDB and HIRB could be important determinants of success in highest-level soccer. Indeed, such speculations can be directly confirmed by our results. We observed significant correlations between TDB and HIRB and successful actions, shots or targets, successful passes, accurate key passes and successful high pressings (all moderate to very strong and correlations), confirming an association between specific TP and TDB/HIRB. In the end, all herein discussed actually supports the results of previous studies, which highlight that RP with ball possession may have a greater influence on success in soccer than RP without ball possession [20, 40, 41].

There were some potential limitations of the current study. First, we did not analyze all matches from the group stage of the UCL (i.e., we only noted 20 randomly selected matches). However, this is a very common obstacle in studies involving players which compete at the highest level of soccer [41, 42]. Furthermore, situational factors such as team and opposition quality, match location or match outcome, which may influence RP in national competitions [13, 17, 43], were not considered in the current study. However, very recent study demonstrated small influence of such situational factors on RP in UCL matches [44]; therefore, influence on results in current study may be negligible. Future research should analyse greater number of RP variables (i.e., distance covered in all speed zones, accelerations, decelerations, metabolic power), considering range of situational factors.

## Conclusion

The teams’ TP, which are considered essential for match success in soccer, were not associated with TD and HIR. Therefore, the results of this investigation suggest that teams’ overall physical performance does not influence success in soccer at the highest level. However, it must be emphasized that herein we studied highest-level soccer players and it is clear that their overall RP is already at highest possible level. Therefore, any conclusion regarding the eventual non-importance of the RP in soccer may be specific to the level investigated in the present study (i.e. UCL).

On the other hand, significant associations between TDB/HIRB and specific TP indicate that teams’ physical performance when in ball possession (i.e., in attacking phase of game) may be important determinant of success in highest-level soccer. Namely, covering greater total and high intensity running distance in attacking phase of game will create more of free spaces on the pitch, allowing more frequent execution of successfully performed technical and tactical solutions by co-players from the team. These findings should form the basis of training methodology for physical conditioning in highest-level soccer teams, encouraging soccer coaches to design exercises which provoke such team behavior in training programs.

In particular, in highest-level soccer, physical conditioning exercises with the ball should be more appropriate than traditional physical training without the ball. Most specifically, soccer coaches should combine physical conditioning exercises with fundamental technical performance such as passing, shooting or crossing, as well as tactical performance such as creating goal chances, successful high pressings and entrances to the opponent’s box. Such training exercises should be carried out on spaces that allow players to develop a high intensity of running, what will at the same time contribute to overcoming higher values of the total distance covered. Translating these exercises from training into the matches may ultimately have important impact on achieving greater success during the matches in highest-level of soccer.

## Data Availability

The datasets generated and/or analysed during the current study are not publicly available due the copyright of InStat Limited (Limerick, Republic of Ireland) but are available from the corresponding author on reasonable request.

## Abbreviations

RP: Running performance
TP: Technical-tactical performance
UCL: UEFA Champions League
TD: Total distance
HIR: High-intensity running
TDB: Total distance when in ball possession
HIRB: High-intensity running when in ball possession
L-TD: Teams covering low total distance
H-TD: Teams covering high total distance
L-HIR: Teams covering low high-intensity running
H-HIR: Teams covering high high-intensity running
L-TDB: Teams covering low total distance with ball in possession
H-TDB: Teams covering high total distance with ball in possession
L-HIR: Teams covering low high-intensity running with ball in possession
H-HIR: Teams covering high high-intensity running with ball in possession

## References

[1] Yi Q, Gómez MA, Wang L, Huang G, Zhang H, Liu H (2019). Technical and physical match performance of teams in the 2018 FIFA World Cup: effects of two different playing styles. J Sports Sci.

[2] Longo UG, Sofi F, Candela V, Risi Ambrogioni L, Pagliai G, Massaroni C (2021). The influence of athletic performance on the highest positions of the final ranking during 2017/2018 Serie A season. BMC Sports Sci Med Rehabil.

[3] Sarmento H, Marcelino R, Anguera MT, CampaniÇo J, Matos N, LeitÃo JC (2014). Match analysis in football: a systematic review. J Sports Sci.

[4] Zhou C, Calvo AL, Robertson S, Gómez M-Á (2021). Long-term influence of technical, physical performance indicators and situational variables on match outcome in male professional Chinese soccer. J Sports Sci.

[5] Rampinini E, Impellizzeri FM, Castagna C, Coutts AJ, Wisløff U (2009). Technical performance during soccer matches of the Italian Serie A league: effect of fatigue and competitive level. J Sci Med Sport.

[6] Andrzejewski M, Oliva-Lozano JM, Chmura P, Chmura J, Czarniecki S, Kowalczuk E (2022). Analysis of team success based on match technical and running performance in a professional soccer league. BMC Sports Sci Med Rehabil.

[7] Goto H, Saward C (2020). The running and technical performance of U13 to U18 elite Japanese soccer players during match play. J Strength Cond Res.

[8] De Albuquerque FL, Brito MA, Muñoz PM, Pérez DIV, Kohler HC, Aedo-Muñoz EA (2022). Match running performance of Brazilian professional soccer players according to tournament types. Montenegrin J Sports Sci Med.

[9] Carling C (2013). Interpreting physical performance in professional soccer match-play: should we be more pragmatic in our approach?. Sports Med.

[10] Trewin J, Meylan C, Varley MC, Cronin J (2017). The influence of situational and environmental factors on match-running in soccer: a systematic review. Sci Med Footb.

[11] Aquino R, Gonçalves LG, Galgaro M, Maria TS, Rostaiser E, Pastor A (2021). Match running performance in Brazilian professional soccer players: comparisons between successful and unsuccessful teams. BMC Sports Sci Med Rehabil.

[12] Bloomfield J, Polman R, O’Donoghue P (2005). Effects of score-line on intensity of play in midfield and forward players in the FA Premier League. J Sports Sci.

[13] Castellano J, Blanco-Villaseñor A, Alvarez D (2011). Contextual variables and time-motion analysis in soccer. Int J Sports Med.

[14] Lago-Peñas C (2012). The role of situational variables in analysing physical performance in soccer. J Hum Kinet.

[15] Moalla W, Fessi MS, Makni E, Dellal A, Filetti C, Di Salvo V (2018). Association of physical and technical activities with partial match status in a soccer professional team. J Strength Cond Res.

[16] Barrera J, Sarmento H, Clemente FM, Field A, Figueiredo AJ (2021). The effect of contextual variables on match performance across different playing positions in professional Portuguese soccer players. Int J Environ Res Public Health.

[17] Aquino R, Carling C, Vieira LHP, Martins G, Jabor G, Machado J (2020). Influence of situational variables, team formation, and playing position on match running performance and social network analysis in Brazilian professional soccer players. J Strength Cond Res.

[18] García-Unanue J, Pérez-Gómez J, Giménez J-V, Felipe JL, Gómez-Pomares S, Gallardo L (2018). Influence of contextual variables and the pressure to keep category on physical match performance in soccer players. PLoS ONE.

[19] Di Salvo V, Gregson W, Atkinson G, Tordoff P, Drust B (2009). Analysis of high intensity activity in Premier League soccer. Int J Sports Med.

[20] Hoppe M, Slomka M, Baumgart C, Weber H, Freiwald J (2015). Match running performance and success across a season in German Bundesliga soccer teams. Int J Sports Med.

[21] Asian Clemente JA, Requena B, Jukic I, Nayler J, Hernández AS, Carling C (2019). Is physical performance a differentiating element between more or less successful football teams?. Sports.

[22] Castellano J, Casamichana D, Lago C (2012). The use of match statistics that discriminate between successful and unsuccessful soccer teams. J Hum Kinet.

[23] Modric T, Versic S, Jelicic M (2022). Monitoring technical performance in the UEFA Champions League: differences between successful and unsuccessful teams. Montenegrin J Sports Sci Med.

[24] Konefał M, Chmura P, Zając T, Chmura J, Kowalczuk E, Andrzejewski M (2019). Evolution of technical activity in various playing positions, in relation to match outcomes in professional soccer. Biol Sport.

[25] Lago-Peñas C, Lago-Ballesteros J, Rey E (2011). Differences in performance indicators between winning and losing teams in the UEFA Champions League. J Hum Kinet.

[26] Yi Q, Gómez-Ruano M, Liu H, Zhang S, Gao B, Wunderlich F (2020). Evaluation of the technical performance of football players in the UEFA Champions League. Int J Environ Res Public Health.

[27] Lorenzo-Martínez M, Padrón-Cabo A, Rey E, Memmert D (2020). Analysis of physical and technical performance of substitute players in professional soccer. Res Q Exerc Sport.

[28] Yang G, Leicht AS, Lago C, Gómez M-Á (2018). Key team physical and technical performance indicators indicative of team quality in the soccer Chinese super league. Res Sports Med.

[29] Collet C (2013). The possession game? A comparative analysis of ball retention and team success in European and international football, 2007–2010. J Sports Sci.

[30] Lago-Peñas C, Lago-Ballesteros J, Dellal A, Gómez M (2010). Game-related statistics that discriminated winning, drawing and losing teams from the Spanish soccer league. J Sports Sci Med.

[31] Kempe M, Vogelbein M, Memmert D, Nopp S (2014). Possession vs. direct play: evaluating tactical behavior in elite soccer. Int J Sports Sci.

[32] Kubayi A (2021). Position-specific physical and technical demands during the 2019 COPA America Football tournament. S Afr J Sports Med.

[33] Liu H, Yi Q, Giménez J-V, Gómez M-A, Lago-Peñas C (2015). Performance profiles of football teams in the UEFA Champions League considering situational efficiency. Int J Perform Anal Sport.

[34] Henderson MJ, Fransen J, McGrath JJ, Harries SK, Poulos N, Coutts AJ (2019). Situational factors affecting rugby sevens match performance. Sci Med Footb.

[35] Batterham AM, Hopkins WG (2006). Making meaningful inferences about magnitudes. Int J Sports Physiol Perform.

[36] Taylor R (1990). Interpretation of the correlation coefficient: a basic review. J Diagn Med Sonogr.

[37] Liu H, Hopkins WG, Gómez MA (2016). Modelling relationships between match events and match outcome in elite football. Eur J Sport Sci.

[38] Ali A (2011). Measuring soccer skill performance: a review. Scand J Med Sci Sports.

[39] da Costa IT, da Silva JMG, Greco PJ, Mesquita I (2009). Tactical principles of Soccer: concepts and application. Motriz.

[40] Brito Souza D, López-Del Campo R, Blanco-Pita H, Resta R, Del Coso J (2020). Association of match running performance with and without ball possession to football performance. Int J Perform Anal Sport.

[41] Modric T, Versic S, Drid P, Stojanovic M, Radzimiński Ł, Bossard C (2021). Analysis of running performance in the offensive and defensive phases of the game: is it associated with the team achievement in the UEFA Champions League?. Appl Sci.

[42] Bradley PS, Carling C, Archer D, Roberts J, Dodds A, Di Mascio M (2011). The effect of playing formation on high-intensity running and technical profiles in English FA Premier League soccer matches. J Sports Sci.

[43] Jerkovic Z, Modric T, Versic S (2022). Analysis of the associations between contextual variables and match running performance in Croatian First Division Soccer. Sport Mont.

[44] Modric T, Versic S, Stojanovic M, Chmura P, Andrzejewski M, Konefał M (2022). Factors affecting match running performance in elite soccer: analysis of UEFA Champions League matches. Biol Sport.

